# A Cross-Sectional Study on Socioeconomic Systems Supporting Outpatients With Parkinson’s Disease in Japan

**DOI:** 10.2188/jea.JE20150081

**Published:** 2016-04-05

**Authors:** Aiko Matsushima, Akihisa Matsumoto, Fumio Moriwaka, Sanae Honma, Kazunori Itoh, Keiko Yamada, Shun Shimohama, Hirofumi Ohnishi, Junichi Matsushima, Mitsuru Mori

**Affiliations:** 1 Department of Public Health, Sapporo Medical University School of Medicine, Sapporo, Japan; 1 札幌医科大学医学部公衆衛生学講座; 2 Department of Neurology, Jozankei Hospital, Sapporo, Japan; 2 定山渓病院神経難病センター神経内科; 3 Hokuyukai Neurological Hospital, Sapporo, Japan; 3 北祐会神経内科病院; 4 Iwamizawa Neurological Medical Clinic, Iwamizawa, Hokkaido, Japan; 4 いわみざわ神経内科・内科クリニック; 5 Department of Neurology, Sapporo Medical University School of Medicine, Sapporo, Japan; 5 札幌医科大学医学部神経内科学講座; 6 Matsushima Oto-laryngeal and Dizziness Clinic, Sapporo, Japan; 6 まつしま耳鼻咽喉科クリニック

**Keywords:** Parkinson’s disease, medical payment, cross-sectional study, パーキンソン病, 医療費, 断面研究

## Abstract

**Objectives:**

We conducted a cross-sectional study to evaluate the socioeconomic systems supporting outpatients with Parkinson’s disease (PD) in Japan.

**Methods:**

The study was performed in 2013 at two private hospitals and one clinic in Hokkaido Prefecture, Japan. A survey was conducted with 248 consecutive PD patients, and the data from 237 PD outpatients were analyzed after excluding 11 patients who did not meet inclusion criteria. Monthly medical and transportation payments as a PD outpatient were selected as outcome variables, and their association with various explanatory variables, such as utilization of support systems for PD outpatients, were evaluated using logistic regression model analysis.

**Results:**

After controlling for potential confounding variables, the utilization of the system providing financial aid for treatment for patients with intractable disease was significantly inversely associated with monthly medical payment among PD outpatients (OR 0.46; 95% CI, 0.22–0.95). Experience of hospital admission for PD treatment was significantly positively associated with monthly transportation payment (OR 4.74; 95% CI, 2.18–10.32). Monthly medical payment was also significantly positively associated with monthly transportation payment (OR 4.01; 95% CI, 2.23–7.51).

**Conclusions:**

Use of Japanese public financial support systems may be associated with reductions in medical payments for PD outpatients. However, those systems may not have supported transportation payments, and higher transportation payments may be associated with an increased risk of hospitalization.

## Abstract (ja)

**目的：:**

日本におけるパーキンソン病の外来通院患者を支援する社会経済的制度を評価するために断面研究を行った。

**方法：:**

本研究は、2013年に北海道内の2つの病院と1つの診療所で実施された。連続抽出した248人のパーキンソン病の外来通院患者に対して調査が行われ、除外基準に該当した11を除く237人のデータが解析された。毎月の通院医療費、および、毎月の通院交通費を目的変数として、パーキンソン病患者を支援する制度などの種々な説明変数との関連性をロジスティック回帰モデル分析によって検討し、オッズ比（OR）とその95%信頼区間（95%CI）を算出した。

**結果：:**

交絡要因となる可能性のある変数を調整した解析で、特定疾患医療費助成制度の利用と通院医療費との間に、有意な負の関連がみられた（OR=0.46, 95%CI 0.22-0.95, P=0.035）。入院の経験と通院交通費との間に、有意な正の関連がみられた（OR=4.74, 95%CI 2.18-10.32, P<0.001）。また、通院医療費と通院交通費との間に、有意な正の関連がみられた（OR=4.01, 95%CI 2.23-7.51, P<0.001）。

**結論：:**

パーキンソン病の患者の通院医療費に対する日本の公的な経済的支援制度は、患者の通院医療費の負担軽減と関連していることが示された。しかし、それらの制度は患者の通院交通費の負担軽減とは関連していなかった。そして、通院交通費が高いことは入院するリスクと関連することが示唆された。

## INTRODUCTION

Parkinson’s disease (PD) is one of the most common progressive and disabling neurological disorders throughout the world. The prevalence of PD in Japan has been reported to be about 95 per 100 000 in 1993 in Hokkaido^1^ and 175 per 100 000 in 2005 in Kochi^2^ and is suggested to be lower in Japan than in Western countries.^1^^,^^2^ According to studies in Japan, the quality of life (QoL) of PD patients is lower than in the general population,^3^ and QoL decreases with increasing severity of PD.^4^ The survival rate of PD patients was also reported to be lower than that of the general population.^5^

Finding employment is difficult for PD patients because of physical impairment, and consequently, their incomes tend to be reduced,^6^ medical costs for the treatment of PD as well as for daily care tend to be relatively high.^7^^–^^9^ Elderly patients with PD are reported to have 84% greater total expenditures compared to those without PD in the United States.^8^ PD patients have been reported to use significantly more health care services across all categories and pay significantly more out of pocket for their medical services than other elderly individuals.^10^ PD has also been shown to have a chronic course with growing disability and considerable socioeconomic burden in Germany, as well.^11^

As briefly explained by Murata et al,^12^ Japan has maintained a nationwide social health insurance system based on the German social health insurance model, and Japanese citizens can receive services from any physician or hospital. Although every patient pays between 10% and 30% of the total medical charges (depending on age class as defined in the public health insurance system), more subjects with low income reported that they had stopped or postponed necessary medical care in the past year.^12^

In addition to the social health insurance system, there are three kinds of public financial support systems for PD patients: a system for patients with intractable diseases to receive financial aid for treatment,^13^ a long-term-care insurance system,^14^^–^^17^ and a system for patients with physical disability certificates.^18^ PD patients are allowed to utilize these systems depending on the stage of their illness and the degree of their disability. However, there are few studies assessing what kind of PD patients need the most financial aid and evaluating whether these three systems successfully support PD patients in receiving financial aid for medical costs and other payments related to health problems. We therefore conducted a cross-sectional study to clarify these issues in PD patients.

## METHODS

The present cross-sectional study was conducted among PD outpatients from February to October in 2013 at two private hospitals and one clinic in Hokkaido Prefecture, Japan. These three medical institutions are famous for treating PD patients in Hokkaido, and each institution had at least two practicing physicians with a certificate of specialty in neurology. The inclusion criterion consisted of outpatients being treated for PD at these three medical institutions. The exclusion criteria consisted of patients who were institutionalized in a nursing home or patients being supported by the Livelihood Protection Law. Among consecutive PD outpatients of the three medical institutes, written informed consent was obtained from every participant. A structured questionnaire was completed through a face-to-face interview with a public health nurse.

The questionnaire included the following items: six items about personal characteristics, such as age, sex, working status, size of household, annual income, and body mass index (BMI; as an index for nutritional deficiency^19^); four items about clinical status, such as time since PD onset, time since PD diagnosis, Hoehn and Yahr stage, and degree of dysphagia (measured with 15 questions developed by Manor et al^20^^,^^21^); six items about medical care, such as monthly medical payments, monthly transportation payments, hospital admission for PD treatment, and utilization of the systems for patients with intractable disease receiving financial aid for treatment, long-term care insurance, and patients with physical disability certificates.

Monthly medical payments and monthly transportation payments were selected as outcome variables. In the questionnaire, both were categorized into six classes: 0 yen, <5000 yen, 5000–9999 yen, 10 000–14 999 yen, 15 000–19 999 yen and ≥20 000 yen (currently, 1 dollar is approximately equal to 120 yen). However, we combined some classes with low frequencies and created three new classes for a more reliable analysis: 0 yen, <5000 yen, and ≥5000 yen. Similarly, the subjects were divided into three classes by monthly transportation payments: <5000 yen, 5000–9999 yen, and ≥10 000 yen.

Among the explanatory variables, age, time since PD onset, and time since PD diagnosis were categorized into two or three classes with reference to their mean values. Hoehn and Yahr stage was divided into two classes using stage 3 as a cut-off point for severity. The degree of dysphagia was also divided into two classes using a score of 11 as a cut-off point for positivity in dysphagia.^20^^,^^21^ BMI was categorized into three classes according to the Japanese standards of obesity: <18.5, 18.5–24.9, and ≥25.0.

Because both of the outcome variables were ordinal variables with three-level categories, univariate analysis was conducted with the Kruscal-Wallis test, and multivariable analysis was performed using the ordinal logistic model or proportional odds model^22^ to adjust for potential confounding variables found to have a significant relationship in the univariate analysis. Adjusted odds ratios (ORs) and their 95% confidence intervals (CIs) were calculated using the model. The proportional odds model is reported to be derived by assuming that the outcome variable was obtained by categorizing some type of continuous variable.^22^ Similar to the simple logistic regression model, an obtained OR shows the strength of the association between the outcome and explanatory variables. Generally speaking, the association is thought to be relatively strong in terms of economical considerations if the OR is more than 1.5. The SAS System version 9.4 (SAS Institute, Tokyo, Japan) was utilized for these analyses. The significance level was set at 5%, and two-sided tests were conducted. This study was approved by the Institutional Review Board of Sapporo Medical University.

## RESULTS

Of the 251 consecutive PD outpatients from the three medical institutes, written informed consent was obtained from 248 (98.8%). Excluding 11 patients that met the exclusion criteria, the data of 237 outpatients were analyzed. The average (standard deviation) age of the 237 PD outpatients at the time of survey and disease onset were 71.2 (8.4) years, and 62.9 (10.3) years, respectively. Among them, 83 subjects were male and 154 subjects were female (male:female ratio, 1:1.86).

Table 1 shows the associations of various factors with monthly medical payment divided into three classes. Time since PD onset (*P* = 0.013) and time since PD diagnosis (*P* = 0.014) were significantly inversely associated with monthly medical payment. A Hoehn and Yahr stage ≥3 was significantly inversely associated with monthly medical payment (*P* = 0.003). Utilization of the system for patients with intractable disease receiving financial aid for treatment was significantly inversely associated with monthly medical payment (*P* < 0.001). Similarly, utilization of a long-term care insurance system (*P* = 0.001) and utilization of the system for patients with physical disability certificates (*P* = 0.029) were significantly inversely associated with monthly medical payment. Lower annual income was significantly associated with lower monthly medical payment (*P* = 0.041).

**Table 1.** Association of various factors with monthly medical payment

Item	Content	None		<5000 yen		≥5000 yen		*P* value^a^
		
		*n*	%	*n*	%	*n*	%	
Age	<65 years	5	20.7	28	19.4	16	26.6	0.607
	65–74 years	8	31.0	65	44.5	23	35.9	
	≥75 years	13	48.3	55	36.1	24	37.5	
Sex	Male	6	23.1	53	35.8	24	38.1	0.272
	Female	20	76.9	95	64.2	39	61.9	
Time since PD onset	<8.0 years	10	38.5	80	54.0	42	66.7	0.013
	≥8.0 years	16	61.5	68	46.0	21	33.3	
Time since PD diagnosis	<5.0 years	12	48.0	79	53.4	44	71.0	0.014
	≥5.0 years	13	52.0	69	46.6	18	29.0	
Hoehn and Yahr stage	<3	5	19.2	64	43.2	35	55.6	0.003
	≥3	21	80.8	84	56.8	28	44.4	
Degree of dysphagia	<11	18	69.2	105	71.4	49	77.8	0.311
	≥11	8	30.8	43	28.6	15	22.2	
BMI	<18.5	3	11.5	23	15.5	7	11.1	0.886
	18.5–24.9	16	61.5	96	64.9	42	66.7	
	≥25.0	7	26.9	29	19.6	14	22.2	
Employed	No	24	92.3	135	91.2	55	87.3	0.635
	Yes	2	7.7	13	8.8	8	12.7	
Single households	No	23	88.5	125	84.5	57	90.5	0.480
	Yes	3	11.5	23	15.5	6	9.5	
Utilization of system for patients with intractable disease receiving financial aid for treatment	No	3	11.5	43	29.1	31	49.2	<0.001
	Yes	23	88.5	105	70.9	32	50.8	
Utilization of long-term care insurance system	No	10	38.5	80	54.0	46	73.0	0.001
	Yes	16	61.5	68	46.0	17	27.0	
Utilization of system for patients with physical disability certificate	No	11	42.3	98	66.2	45	71.4	0.029
	Yes	15	57.7	50	33.8	18	28.6	
Hospital admission for PD treatment	No	17	65.4	93	62.8	47	74.6	0.179
	Yes	9	34.6	55	37.2	16	25.4	
Annual income	<2.0 milion yen	15	67.7	50	34.7	19	30.2	0.041
	2.0–3.9 milion yen	10	38.5	72	50.0	30	47.6	
	≥4.0 million yen	1	3.9	22	15.3	14	22.2	

BMI, body mass index; PD, Parkinson’s disease.

^a^Using the Kruskal-Wallis test.

As shown in Table 2, even after adjusting for potential confounding variables found to have a significant relationship in the univariate analysis (see Table 1), the utilization of the system for patients with intractable disease receiving financial aid for treatment was significantly inversely associated with monthly medical payment (OR 0.46; 95% CI, 0.22–0.95).

**Table 2.** Multivariable-adjusted odds ratios^a^ of factors associated with monthly medical payment

Item	OR	95% CI
Time since onset	0.80	0.43–1.46
Hoehn and Yahr stage ≥3	0.75	0.39–1.43
Utilization of system for patients with intractable disease receiving financial aid for treatment	0.46	0.22–0.95
Utilization of long-term care insurance system	0.66	0.34–1.28
Utilization of system for patients with physical disability certificate	0.77	0.39–1.50
Annual income	1.42	0.95–2.12

CI, confidence interval; OR, odds ratio; PD, Parkinson’s disease.

^a^Monthly costs of transportation payment as PD patient were also involved in this analysis, but its odds are not shown here because the same results are shown in Table 4.

Table 3 shows the associations of various factors with monthly transportation payment divided into three classes. Utilization of the system for patients with intractable disease receiving financial aid for treatment was significantly positively associated with monthly transportation payment (*P* = 0.029). Utilization of the system for patients with physical disability certificates was significantly positively associated with monthly transportation payment (*P* = 0.012). Likewise, hospital admission for PD treatment was significantly positively associated with monthly transportation payment (*P* < 0.001). Monthly medical payment was significantly positively associated with monthly transportation payment (*P* < 0.001). Other variables, such as age, sex, time since PD onset, Hoehn and Yahr stage, degree of dysphagia, and annual income, were not associated with monthly transportation payment.

**Table 3.** Association of various factors with monthly transportation payment

Item	Content	<5000 yen		5000– 10 000 yen		≥10 000 yen		*P* value^a^
		
		*n*	%	*n*	%	*n*	%	
Age	<65 years	40	23.3	5	11.6	3	15.8	0.197
	65–74 years	70	40.7	16	37.2	9	47.4	
	≥75 years	62	36.0	22	51.2	7	36.8	
Sex	Male	59	34.3	17	39.5	5	26.3	0.981
	Female	113	65.7	26	60.5	14	73.7	
Time since PD onset	<8.0 years	99	57.6	21	48.8	11	57.9	0.481
	≥8.0 years	73	42.4	22	51.2	8	42.1	
Time since PD diagnosis	<5.0 years	92	54.1	25	58.1	15	79.0	0.106
	≥5.0 years	78	45.9	18	41.9	4	21.0	
Hoehn and Yahr stage	<3	77	44.8	46	44.2	20	36.8	0.648
	≥3	95	55.2	19	55.8	7	63.2	
Degree of dysphagia	<11	126	73.7	30	69.8	13	68.4	0.508
	≥11	45	26.3	13	30.2	6	31.6	
BMI	<18.5	26	15.1	3	7.0	2	10.5	0.363
	18.5–24.9	111	64.5	31	72.1	11	57.9	
	≥25.0	35	20.4	9	20.9	6	31.6	
Employed	No	153	90.0	42	97.7	17	89.5	0.202
	Yes	19	11.0	1	2.3	2	10.5	
Single households	No	150	87.2	36	83.7	16	84.2	0.522
	Yes	22	12.8	7	16.3	3	15.8	
Utilization of system for patients with intractable disease receiving financial aid for treatment	No	63	36.6	12	27.9	2	10.5	0.029
	Yes	109	63.4	31	72.1	17	89.5	
Utilization of long-term care insurance system	No	100	58.1	25	58.1	9	47.4	0.578
	Yes	72	41.9	18	41.9	10	52.6	
Utilization of system for patients with physical disability certificate	No	120	69.8	22	51.2	10	52.6	0.012
	Yes	52	30.2	21	48.8	9	47.4	
Hospital admission for PD treatment	No	128	74.4	20	46.5	6	31.6	<0.001
	Yes	44	25.6	23	53.5	13	68.4	
Monthly medical payment	None	22	12.8	2	4.7	1	5.3	<0.001
	<5000 yen	116	67.4	19	44.2	11	57.9	
	≥5000 yen	34	19.8	43	18.4	7	36.8	
Annual income	<2.0 milion yen	66	39.3	15	34.9	2	10.5	0.160
	2.0–3.9 milion yen	76	45.2	24	55.8	11	57.9	
	≥4.0 million yen	26	15.5	4	9.3	6	31.6	

BMI, body mass index; PD, Parkinson’s disease.

^a^Using the Kruskal-Wallis test.

As shown in Table 4, even after adjusting for potential confounding variables found to have a significant relationship in the univariate analysis (see Table 3), hospital admission for PD treatment was significantly positively associated with monthly transportation payment (OR 4.74; 95% CI, 2.18–10.32). Amount of monthly medical payment was also significantly positively associated with monthly transportation payment (OR 4.01; 95% CI, 2.23–7.51).

**Table 4.** Multivariable-adjusted odds ratios of factors associated with monthly transportation payment among PD outpatients

Item	OR	95% CI
Utilization of system for patients with intractable disease receiving financial aid for treatment	1.68	0.73–3.87
Utilization of system for patients with physical disability certificate	1.02	0.48–2.16
Hospital admission for PD treatment	4.74	2.18–10.32
Monthly medical payment	4.01	2.23–7.51

CI, confidence interval; OR, odds ratio; PD, Parkinson’s disease.

## DISCUSSION

The system for patients with an intractable disease receiving financial aid for treatment started for PD patients in 1978,^13^ and utilization of this system was significantly inversely associated with monthly medical payment in our study, even after adjustment for potential confounding variables. This system might be effective in the reduction of payments among PD patients, especially in advanced PD patients with higher medical payments.

The Hoehn and Yahr stage has been shown to be significantly positively associated with medical expenditure in previous studies conducted outside of Japan, such as those conducted in the United Kingdom,^23^ China,^24^ Finland,^25^ the Czech Republic,^26^ Germany,^27^ and the United States.^28^ However, Hoehn and Yahr stage was inversely associated with monthly medical payment in our study in Japan, although the significance disappeared after controlling for potential confounding variables. This fact might indicate that PD patients with more severe Hoehn and Yahr stages are more likely to be financially supported by the public support system in Japan.

The long-term-care insurance system started in 2000,^14^ and utilization of this system was significantly inversely associated with monthly medical payment, although the significance disappeared after controlling for potential confounding variables. Because this system has been shown to be effective in increasing utilization of care services, such as frequency of nurses visiting PD patients at home,^16^ this system might be effective in reducing medical payments among PD outpatients by providing for care at home without financial conflict.

Although the system for patients with an intractable disease receiving financial aid for treatment does not support transportation costs, financial support for transportation has been provided through the system for patients with physical disability certificates.^29^ However, these reimbursements do not seem to be enough, because using this system was not associated with reduced transportation payment in this study. Further, higher transportation payment was shown to be associated with increased likelihood to be hospitalized as a PD inpatient in this study. Transportation problems have been reported to delay and discourage hospital attendance.^30^ A significantly higher frequency of transportation problems was also reported in the elderly, especially those with lower incomes.^12^ Patients who receive home oxygen therapy^31^ and patients with severe congenital heart disease^32^ have claimed that they have been mostly unsatisfied with the high costs of transportation. Since the burden of transportation payments among PD patients has been scarcely reported in Japan, further investigation is required.

Several limitations should be discussed. First, we were unable to determine a causal relationship due to the cross-sectional design. Second, our study subjects were recruited from three medical institutions in Hokkaido that are known for their quality of care in PD treatment, so their social supports for treating PD patients may be better than at other institutions. Therefore, our results may not be representative of all Japanese PD patients.

Because our data were obtained through the questionnaire, measurements of monthly medical payments as well as monthly transportation payments were not precise, but approximate. The data depended on the PD outpatient and for example, by whether they included medical payments other than PD treatments in the monthly medical payment or not, whether they included payment for taxi usage in the monthly transportation payment, and whether they answered monthly payment using an average or for a specific month. Furthermore, we did not reference medical payment records from each institution, although such a strategy would more accurately estimate the amounts of medical payments. Finally, our categorizations of the two outcome variables may not have been appropriate, because the obtained results were not uniformly distributed among the categories. Further study with a more refined study design is necessary to overcome these limitations.

In conclusion, Japanese public financial supporting systems may be associated with reduction in out-of-pocket medical payments among PD outpatients. However, the existing systems may not adequately cover transportation costs, and higher transportation payments may be associated with an increased risk of hospitalization.

## ONLINE ONLY MATERIAL

Abstract in Japanese.
