# Supplementary material for: A Cross-Sectional Study on Socioeconomic Systems Supporting Outpatients With Parkinson’s Disease in Japan
Source: J Epidemiol. 2016 Apr 5;26(4):185–90. doi: 10.2188/jea.JE20150081 (PMC4808685; doi:10.2188/jea.JE20150081)
Supplement: Abstract in Japanese. [file je-26-185-s001.pdf]

タイトル：パーキンソン病の外来通院患者を支援する社会経済的制度に関する横断研究

氏名：松島　愛子[1]、松本　昭久[2]、森若　文雄[3]、本間　早苗[3]、伊藤　和則[4]、
山田　恵子[4]、下濱　俊[5]、大西　浩文[1]、松島　純一[6]、森　　満[1]

所属：[1]札幌医科大学医学部公衆衛生学講座
[2]定山渓病院神経難病センター神経内科
[3]北祐会神経内科病院
[4]いわみざわ神経内科・内科クリニック
[5]札幌医科大学医学部神経内科学講座
[6]まつしま耳鼻咽喉科クリニック

目的：日本におけるパーキンソン病の外来通院患者を支援する社会経済的制度を評価するために断面研究を行った。

方法：本研究は、2013年に北海道内の2つの病院と1つの診療所で実施された。連続抽出した248人のパーキンソン病の外来通院患者に対して調査が行われ、除外基準に該当した11を除く237人のデータが解析された。毎月の通院医療費、および、毎月の通院交通費を目的変数として、パーキンソン病患者を支援する制度などの種々な説明変数との関連性をロジスティック回帰モデル分析によって検討し、オッズ比(OR)とその95%信頼区間(95%CI)を算出した。

結果：交絡要因となる可能性のある変数を調整した解析で、特定疾患医療費助成制度の利用と通院医療費との間に、有意な負の関連がみられた（OR=0.46, 95%CI 0.22-0.95, P=0.035）。入院の経験と通院交通費との間に、有意な正の関連がみられた（OR=4.74, 95%CI 2.18-10.32, P<0.001)。また、通院医療費と通院交通費との間に、有意な正の関連がみられた(OR=4.01, 95%CI 2.23-7.51, P<0.001）。

結論：パーキンソン病の患者の通院医療費に対する日本の公的な経済的支援制度は、患者の通院医療費の負担軽減と関連していることが示された。しかし、それらの制度は患者の通院交通費の負担軽減とは関連していなかった。そして、通院交通費が高いことは入院するリスクと関連することが示唆された。

キーワード：パーキンソン病、医療費、断面研究
